# Neuroscientific explanations and the stigma of mental disorder: a meta-analytic study

**DOI:** 10.1186/s41235-018-0136-1

**Published:** 2018-11-14

**Authors:** Amy Loughman, Nick Haslam

**Affiliations:** 1 Food & Mood Centre, Deakin University, Geelong, VIC 3220 Australia; 2 Melbourne School of Psychological Sciences, University of Melbourne, Parkville, VIC 3010 Australia

**Keywords:** Essentialism, Stigma, Mental disorder, Psychiatric disorder, Brain disease, Blame

## Abstract

Genetic and other biological explanations appear to have mixed blessings for the stigma of mental disorder. Meta-analytic evidence shows that these “biogenetic” explanations reduce the blame attached to sufferers, but they also increase aversion, perceptions of dangerousness, and pessimism about recovery. These relationships may arise because biogenetic explanations recruit essentialist intuitions, which have known associations with prejudice and the endorsement of stereotypes. However, the adverse implications of biogenetic explanations as a set may not hold true for the subset of those explanations that invoke neurobiological causes. Neurobiological explanations might have less adverse implications for stigma than genetic explanations, for example, because they are arguably less essentialist. Although this possibility is important for evaluating the social implications of neuroscientific explanations of mental health problems, it has yet to be tested meta-analytically. We present meta-analyses of links between neurobiological explanations and multiple dimensions of stigma in 26 correlational and experimental studies. In correlational studies, neurobiological explanations were marginally associated with greater desire for social distance from people with mental health problems. In experimental studies, these explanations were associated with greater desire for social distance, greater perceived dangerousness, and greater prognostic pessimism. Neurobiological explanations were not linked to reduced blame in either set of studies. By implication, neurobiological explanations have the same adverse links to stigma as other forms of biogenetic explanation. These findings raise troubling implications about the public impact of psychiatric neuroscience research findings. Although such findings are not intrinsically stigmatizing, they may become so when viewed through the lens of neuroessentialism.

## Significance

Neuroscientific explanations of mental health problems are increasingly prominent in the psychiatric and psychological literature, and they are becoming more widely endorsed by the general public. At the same time, mental health problems continue to be heavily stigmatized and there are few signs that this stigma is abating. It has been argued that biological explanations might play a role in reducing psychiatric stigma, but the evidence to date indicates that they are a double-edged sword, reducing some forms of stigma but exacerbating others. However, no previous studies have examined how the narrower set of neurobiological explanations are linked to stigma, and whether they might have less adverse links to stigma than other forms of biological explanation (e.g., genetic explanations). The present study reports meta-analyses of correlational and experimental studies on this question, and indicates that neurobiological explanations tend to be associated with greater stigma, especially in experimental studies. These findings suggest that laypeople apprehend neuroscientific research findings with an essentialist bias that leads them to ascribe mental health problems to fixed and unchanging pathological essences. The study has implications for how neuroscientific research findings on mental health should be communicated so as to minimize adverse effects on stigma.

## Background

How people respond to neuroscientific explanations is emerging as a dynamic field of research in cognitive psychology. Researchers have explored why these explanations have a particular allure relative to mentalistic explanations (Weisberg, Keil, Goodstein, Rawson, & Gray, 2008), and isolated some of the processes that account for that allure (Hopkins, Weisberg, & Taylor, 2016). Other researchers have explored the role of neuroscientific explanations in moral judgments such as sentencing decisions (Aspinwall, Brown, & Tabery, 2012), how framing behavior concretely versus abstractly influences judgments of the plausibility of neuroscientific explanations for behavior (Kim, Johnson, Ahn, & Knobe, 2017), and how essentialist reasoning may underpin some of these effects (Ahn, Flanagan, Marsh, & Sanislow, 2006). This body of work has vital implications for understanding the public reception of neuroscientific findings.

These issues are especially germane to the field of psychiatry, where neuroscience has become the royal road to understanding mental disorders. Research on the neurobiological dimensions of psychiatric conditions outstrips research on its psychosocial dimensions, and treatments that target brain chemistry are increasingly dominant. With the advent of the National Institute of Mental Health (NIMH) Research Domain Criteria (Cuthbert, 2015), psychiatric classification is shrugging off its reputation for ignoring causation and is making direct reference to underlying neurobiological mechanisms of disorder. Mental health is the second most common domain in which neuroscience receives media coverage (O’Connor, Rees, & Joffe, 2012), and research shows that laypeople increasingly endorsed neurobiological explanations^1^ for mental health problems over the period 1990 to 2006 (Schomerus et al., 2012). These trends suggest that mental disorder is a crucial domain in which people’s responses to neuroscientific explanations can be examined.

The implications of the rising public exposure to psychiatric neuroscience are uncertain. Some critics have lamented the growing “medicalization” of mental disorder (Conrad, 2007) and pointed to the pitfalls of viewing mental disorders as biomedical diseases (Mehta & Farina, 1997). Others have argued that public acceptance of neurobiological and genetic explanations of mental disorders – together commonly referred to as “biogenetic” explanations – should reduce moralistic and punitive responses to sufferers by clarifying that mental health problems are not the result of weak will or bad character. Whatever the merits of these two perspectives, there is evidence that an entirely optimistic reading of the public’s growing endorsement of biogenetic explanations is untenable. The same meta-analysis that tracked growth in biogenetic causal beliefs also revealed that public acceptance of people with schizophrenia declined during the same period, and acceptance of depressed people was unchanged (Schomerus et al., 2012). It is therefore important to determine whether laypeople’s endorsement of biogenetic explanations for mental disorder have positive, negative, or mixed implications for stigma.

Two meta-analyses have addressed this question. Both examined whether the broad class of biogenetic explanations – including genetic, general biological, as well as specifically neurobiological explanations – are associated with public attitudes towards people experiencing mental health problems. Kvaale, Gottdiener, and Haslam (2013) synthesized 28 studies that investigated how experimentally manipulated explanations influenced stigmatizing attitudes, and Kvaale, Haslam, and Gottdiener (2013) reviewed 25 correlational studies of associations between explanations and stigma. Both meta-analyses examined multiple dimensions of stigma – blame directed at sufferers, desire for social distance from them, perceptions of them as dangerous or unpredictable, and pessimism about their recovery – and included studies published prior to November 2012 and October 2011, respectively. There were insufficient studies to allow meaningful comparison of different types of biogenetic explanation (genetic, general biological, or neurobiological), so all types were aggregated.

The two meta-analyses yielded a consistent pattern of findings. The analysis of experimental studies (Kvaale, Gottdiener, & Haslam, 2013) indicated that biogenetic explanations reduced blame, increased perceived dangerousness and prognostic pessimism, and had no effect on social distance. The analysis of correlational studies (Kvaale, Haslam, & Gottdiener, 2013) found that people who endorse biogenetic explanations tend to blame affected persons less for their problems, but perceive them as more dangerous and desire greater social distance from them. No correlational studies addressed prognostic pessimism. The findings of the two meta-analyses therefore supported the view that although biogenetic explanations have the beneficial effect of diminishing moralistic perceptions of people with mental disorders, they have the adverse effects of fostering fear, avoidance, and pessimism.

Reflecting on this evidence that biogenetic explanations of psychopathology are double-edged swords, Haslam and Kvaale (2015) proposed the “mixed-blessings” model of stigma. The model proposes that biogenetic explanations reduce one form of stigma by promoting attributions of personal uncontrollability. The sufferer’s problems are ascribed to a biological abnormality over which they have no control, disarming any tendency to hold them responsible and blameworthy for their unusual behavior and experience. However, the model argues that the same explanations simultaneously increase other forms of stigma by recruiting an essentialist mode of thinking. Research on psychological essentialism indicates that people commonly believe that a deep-seated hidden essence is shared by all members of a category, giving rise to its observable properties and determining its identity. Essentialist beliefs about social categories, which intuit a hidden essence that grounds group membership, often have adverse implications. They have been shown to be associated with prejudice (e.g., Haslam & Levy, 2006), avoidance of outgroups (Williams & Eberhardt, 2008), and endorsement of group stereotypes (Bastian & Haslam, 2006). Researchers have documented essentialist beliefs in laypeople’s thinking about mental disorder (Ahn, Flanagan, Marsh, & Sanislow, 2006; Haslam & Ernst, 2002). Thus, if people understand biogenetic causes of mental disorder categories to be discrete, deep-seated, and unchanging pathological essences, this understanding may have unfortunate consequences (Howell, Weikum, & Dyck, 2011). Because the essence is discrete, the affected person is viewed as categorically different from normality, encouraging social distance. Because the pathological essence is fixed, essentialist thinking implies prognostic pessimism. Finally, because essentialist thinking is associated with stereotype endorsement, people who hold biogenetic explanations of mental disorders are apt to endorse the widespread view that the mentally ill are dangerous and unpredictable.

The mixed-blessings model of psychiatric stigma proposes that biogenetic explanations combine positive (de-stigmatizing) and negative (stigmatizing) implications, the latter due to a tendency for laypeople to understand – or misunderstand – biogenetic causes in an essentialist manner. Such a tendency is highly plausible for genetic causes, where essentialist thinking and its link to prejudice are well-documented. Genes are popularly understood to be discrete, hidden, fixed, and identity-determining to the point where DNA has become a colloquial synonym for essence. Dar-Nimrod and Heine (2011a) have reviewed at length the destructive social implications of genetic essentialism, and Keller (2006) and others have demonstrated its association with racial prejudice. However, genetic causes are only a subset of the broader set of biogenetic causes, and it is not self-evident that other biological causes are understood in equally essentialist ways by laypeople. If they are not understood in this way, these non-genetic biological explanations for mental health problems may not have the adverse implications for stigma predicted by the mixed-blessings model.

Neurobiological explanations – those that invoke causes involving the nervous system – are a particularly important comparison case to genetic explanations. Some writers (Haslam, 2011; Racine, Waldman, Rosenberg, & Illes, 2010) argue that the public sometimes comprehends neurobiological causes in essentialist ways, coining the term “neuroessentialism.” However, although laypeople may understand genes as causally potent essences that are discrete and static, they may understand brain phenomena in less binary and more dynamic ways, encouraged perhaps by popular writing on neural plasticity. Similarly, they may judge genes to be deep, “ultimate” causes whereas neurobiological causes may be judged to operate at a more intermediate level, in between ultimate causes and overt behavior and experience. Thus, when members of the public ascribe a mental disorder to a brain abnormality or a “chemical imbalance” they may not be making an essentialist explanation to the same degree as when they ascribe it to a genetic mutation. As Dar-Nimrod and Heine (2011b) suggest, “it would be interesting and informative to compare the magnitude of essentialist biases between genetic concepts and other potential essence placeholders such as neurological mechanisms” (p.830). The literature has rapidly expanded since the existing meta-analytic study of the correlational relationship between biogenetic explanations and stigma (Kvaale, Haslam, & Gottdiener, 2013), which conducted some preliminary comparisons between neurobiological and genetic explanations, and no quantitative review has addressed the effects on stigma of experimentally inducing neurobiological causal beliefs.

A new meta-analytic review of the literature on the links between neurobiological explanations and stigma is therefore sorely needed. If neurobiological explanations are importantly different from other biogenetic explanations – if they are less essentialist, for example – they may not have the same adverse implications for stigma as those other explanation types, such as explanations invoking genes and heredity. If neurobiological explanations were found not to be associated with greater stigma, in contrast to the findings obtained for biogenetic explanations as a whole, this would provide reassurance that the growing public acceptance of psychiatric neuroscience is unlikely to have negative implications for attitudes towards the mentally ill. If, on the other hand, neurobiological explanations show the same links to stigma as have been demonstrated in past research on biogenetic explanations in general, then that growing public acceptance may have troubling social implications.

The present meta-analytic study therefore aimed to clarify whether neurobiological explanations – those based on popular reception of neuroscientific research – have the same mixed implications for stigma as the broad class of biogenetic explanations. The study partially replicated the two meta-analyses of Kvaale and colleagues. Like the work of Kvaale et al., the present study reports two meta-analyses, one a meta-analysis of studies in which neurobiological explanations were experimentally manipulated and their effects on stigma were observed, and the other a meta-analysis of studies where endorsement of these explanations was correlated with measures of stigma. These two types of study generate effect-size metrics that cannot be directly compared, necessitating two separate meta-analyses, the findings of which are presented separately in the “Results” section. The present study differs from Kvaale, Gottdiener, and Haslam (2013) and Kvaale, Haslam, and Gottdiener (2013) in the primary studies that it meta-analyzed: it only included the subset of the Kvaale et al. studies that specifically examined neurobiological explanations, and also included studies of these explanations that were published in the five or more years since the 2011 and 2012 cutoffs for the Kvaale, Gottdiener, and Haslam (2013) and Kvaale, Haslam, and Gottdiener (2013) investigations. The present study made no predictions about whether neurobiological explanations would have the same or different relationships with stigma as the broader class of biogenetic explanations. If the same pattern of relationships were obtained then neurobiological explanations would have the same problematic implications for stigma as other biogenetic explanations.

## Method

### Systematic review and study selection

Studies included in this review were obtained as part of a larger systematic review of mental disorder stigma (publication in progress), and comprised an updated search and analysis of two articles published in 2013 (Kvaale, Gottdiener, & Haslam, 2013; Kvaale, Haslam, & Gottdiener, 2013). A comprehensive search for relevant articles was conducted in February and updated in October 2017 using the PsycINFO, Medline and PubMed databases. Search terms can be found in “Appendix”. One author (AL) determined eligibility at the title/abstract level, and both authors assessed eligibility from full-text and extracted data. The final sample of 26 articles included 17 that had been included in the Kvaale, Gottdiener, and Haslam (2013), Kvaale, Haslam, and Gottdiener (2013) meta-analyses and 9 new studies.

To be included in the meta-analysis, articles had to (1) be written in English or German, (2) report findings from a plausible and empirically checked experimental manipulation of belief in neurobiological explanations for psychological difficulties or report correlational association(s) between endorsement of neurobiological explanations and stigma in the context of mental disorder, (3) report an effect of neurobiological explanations (e.g., brain disorder/chemical imbalance) on one of the four relevant categories of stigma measures (blame; perceptions of dangerousness; desire for social distance; prognostic pessimism), and (4) report sufficient statistical information for an effect size to be calculated. Studies were excluded if biological explanations were limited to genetic or unspecified biological causes rather than to neurobiological causes. Two studies with explanations that did not explicitly refer to the brain or nervous system but were judged to imply specific effects on neurobiology (i.e., having a nervous breakdown as a result of a “disease … which affected my biochemistry or metabolism” (Mehta & Farina, 1997) and “virus or infection” (Jorm & Griffiths, 2008)) were included. Exclusion of these two studies would have made no difference to the significance levels of findings presented here. Studies were also excluded, if experimental manipulations of neurobiological explanations were combined with other anti-stigma measures, if stigma measures did not specify the nature of the stigma, and if data were not suitable for incorporation into the meta-analysis. Corresponding authors were contacted when the data format was not suitable for meta-analysis.

### Data analysis

Meta-analysis was conducted separately for experimental and correlational studies, and for each of the four categories of stigma measures using Comprehensive Meta-Analysis Version 3 (Borenstein, Hedges, Higgins, & Rothstein, 2014), following the meta-analytic procedures used by Kvaale, Gottdiener, and Haslam (2013), Kvaale, Haslam, and Gottdiener (2013). Hedges’ *g* was the summary measure used for the experimental studies, with effects standardized from independent and repeated measures summary statistics. For the correlational studies, effect size measures were standardized to correlations. For effects originally presented as standardized regression beta weights, conversion to correlations was conducted using the Peterson and Brown (2005) formula. Effect sizes originally presented in odds ratios were converted to Cohen’s *d* and then to correlation *r* as per instructions from Comprehensive Meta-Analysis. A random effects model was used since it presumes different mean effect sizes across studies. Studies were weighted by sample size. Where multiple measures of the same construct were available in a single study, results from each were entered and then averaged. Similarly, data from different diagnostic groups within a single study were averaged to generate one summary statistic, as there were insufficient data to conduct formal subgroup analyses (e.g., stigma associated with depression compared with schizophrenia).

### Study design and measures

The eligible studies measured or presented a variety of neurobiological explanations including brain disease or dysfunction or chemical imbalance (see Table 1). Effects within these studies that pertained to other types of biogenetic explanations (e.g., genes, heredity) were not included in the analyses.

**Table 1:** Types of neurobiological explanations measured or presented in the various studies

Study	Year	Type(s) of explanation	Experimental or correlational
Angermeyer & Matschinger*	2003	Brain disease	Correlational
Angermeyer et al.	2015	Brain disease	Correlational
Angermeyer et al.	2013	Brain disease	Correlational
Arens, Berger, & Lincoln*	2009	Brain disease	Correlational
Aspinwall et al.*	2012	Brain dysfunction	Experimental
Bag, Yilmaz, & Kirpinar*	2006	Brain disease	Correlational
Cheng	2015	Chemical imbalance	Experimental
Deacon & Baird*	2009	Chemical imbalance	Experimental
Dietrich et al.*	2004	Brain disease	Correlational
Dietrich, Matschinger, & Angermeyer*	2006	Brain disease	Correlational
Jorm & Griffiths*	2008	Virus or infection	Correlational
Kemp, Lickel, & Deacon	2014	Chemical imbalance	Experimental
Koschade & Lynd-Stevenson	2011	Chemical imbalance	Correlational
Lam & Salkovskis*	2007	Chemical imbalance and brain dysfunction	Experimental
Lincoln, Arens, Berger, & Rief*	2008	Brain disease and brain damage	Correlational
Luty, Easow, & Mendes	2011	Chemical disturbance	Experimental
Martin, Pescosolido, & Tuch*	2000	Chemical imbalance	Correlational
Martin, Pescosolido, Olafsdottir, & McLeod*	2007	Chemical imbalance	Correlational
Mehta & Farina*	1997	Disease with biochemical effects	Experimental
Meurk, Carter, Partridge, Lucke, & Hall	2014	Brain chemistry	Correlational
Pirutinsky, Rosen, Safran, & Rosmarin*	2010	Chemical imbalance	Correlational
Reavley & Jorm	2014	Composite of genetic and chemical imbalance explanations	Correlational
Rusch, Todd, Bodenhausen, & Corrigan*	2010	Brain disorder caused by changes in brain metabolism	Correlational
Schnittker*	2008	Chemical imbalance	Correlational
Speerforck et al.	2014	Chemical imbalance and brain disease	Correlational
Van’t Veer, Kraan, Drosseart, & Modde*	2006	Brain dysfunction	Correlational

*Studies that were also included in the Kvaale, Gottdiener, and Haslam (2013), Kvaale, Haslam, and Gottdiener (2013) meta-analyses

Operational definitions of the four stigma constructs were as per Kvaale, Gottdiener, and Haslam (2013), as follows. “Blame” refers to any outcome relating to a feeling of responsibility, blame, or anger towards the person with the mental disorder. “Perceived dangerousness” refers to any measure of dangerousness, fear, unpredictability or risk to self/others. “Social distance” refers to unwillingness to enter social relationships with individuals experiencing mental disorder. “Prognostic pessimism” refers to pessimism about chance of recovery, long duration for improvement, or ability of the individual to manage their problems. Outcome measures varied between studies. Where more than one outcome was reported for a single construct within the same study, the results were averaged. This is denoted by an asterisk next to the author names on the forest plots. Mental disorder types included depression, schizophrenia, substance abuse and eating disorders. Samples included university students, general public, and people experiencing mental disorder. Study numbers were too few to undertake subgroup analyses of disorder or sample types.

Experimental studies reviewed here compared ratings of stigma on one or more of the above four stigma constructs following exposure either to a neurobiological explanation or psychosocial explanation of mental disorder. Correlational studies reported on correlations, regression weights, or odds ratios between type of explanation (neurobiological or psychosocial), or the degree to which it was held, and measures of one or more of the four stigma constructs.

## Results

**Fig. 1:**
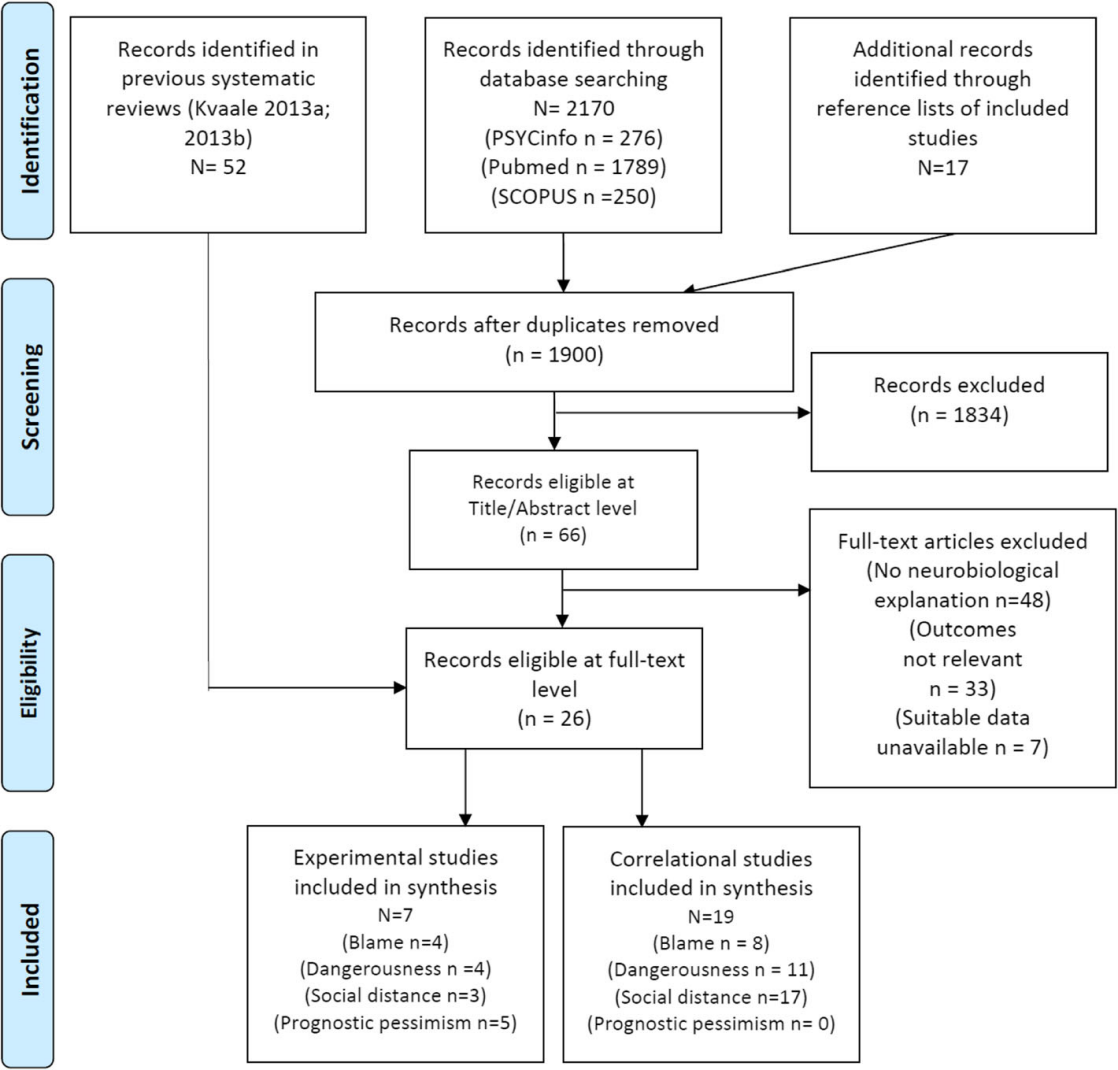
Preferred reporting items for systematic reviews and meta-analyses (PRISMA) flow-chart of systematic review results

A total of 26 studies were found eligible for inclusion in the meta-analysis, including 19 correlational and 7 experimental studies (see Fig. 1).

### Experimental studies

**Fig. 2:**
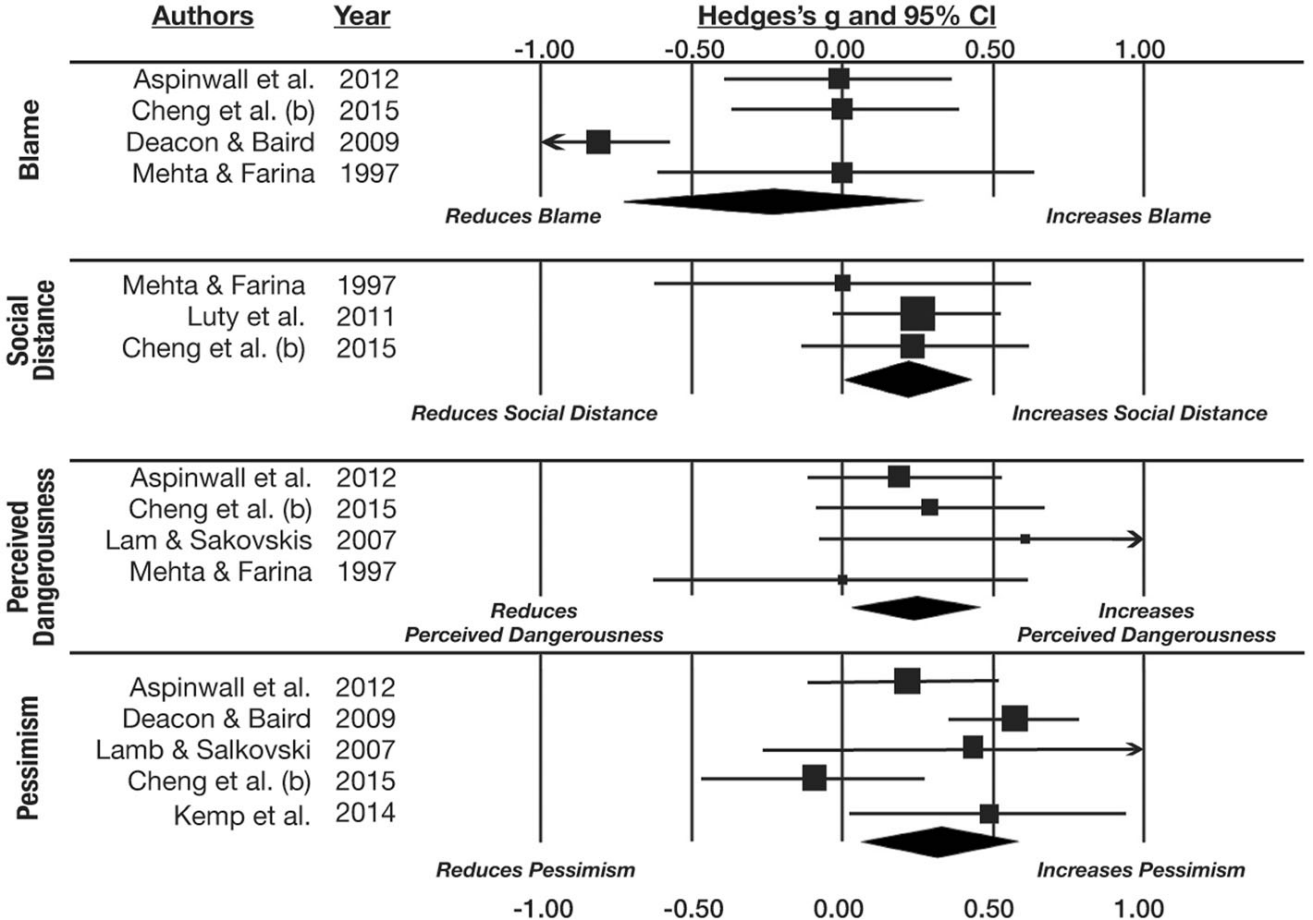
Plots show meta-analyses of the experimental studies

The first set of meta-analyses synthesized the seven experimental studies in which effects of manipulated neurobiological explanations on stigma were examined. Four studies yielded effect seizes for the blame component, three for social distance, four for perceived dangerousness, and five for prognostic pessimism. The findings are presented in Fig. 2 and Table 2. Participants induced to endorse neurobiological explanations for mental health problems did not blame affected persons more or less (*g* = − 0.240, *p* = .349), but they desired significantly more social distance from them (*g* = 0.216, *p* = .045), perceived them as significantly more dangerous (*g* = 0.254, *p* = .02), and were more pessimistic about their recovery (*g* = 0.323, *p* = .018). There were too few studies examining specific conditions to explore whether these moderated the effects.

**Table 2:** Summary of meta-analytic findings from experimental studies

Stigma component	Number of studies	Hedges’ *g* estimate	95% CI - lower	95% CI - upper	*P*
Blame	4	−0.240	−.734	.259	.349
Social distance	3	0.216	.012	.427	.045
Dangerousness	4	0.254	.037	.471	.022
Prognostic pessimism	5	0.323	.057	.589	.018

### Correlational studies

**Fig. 3:**
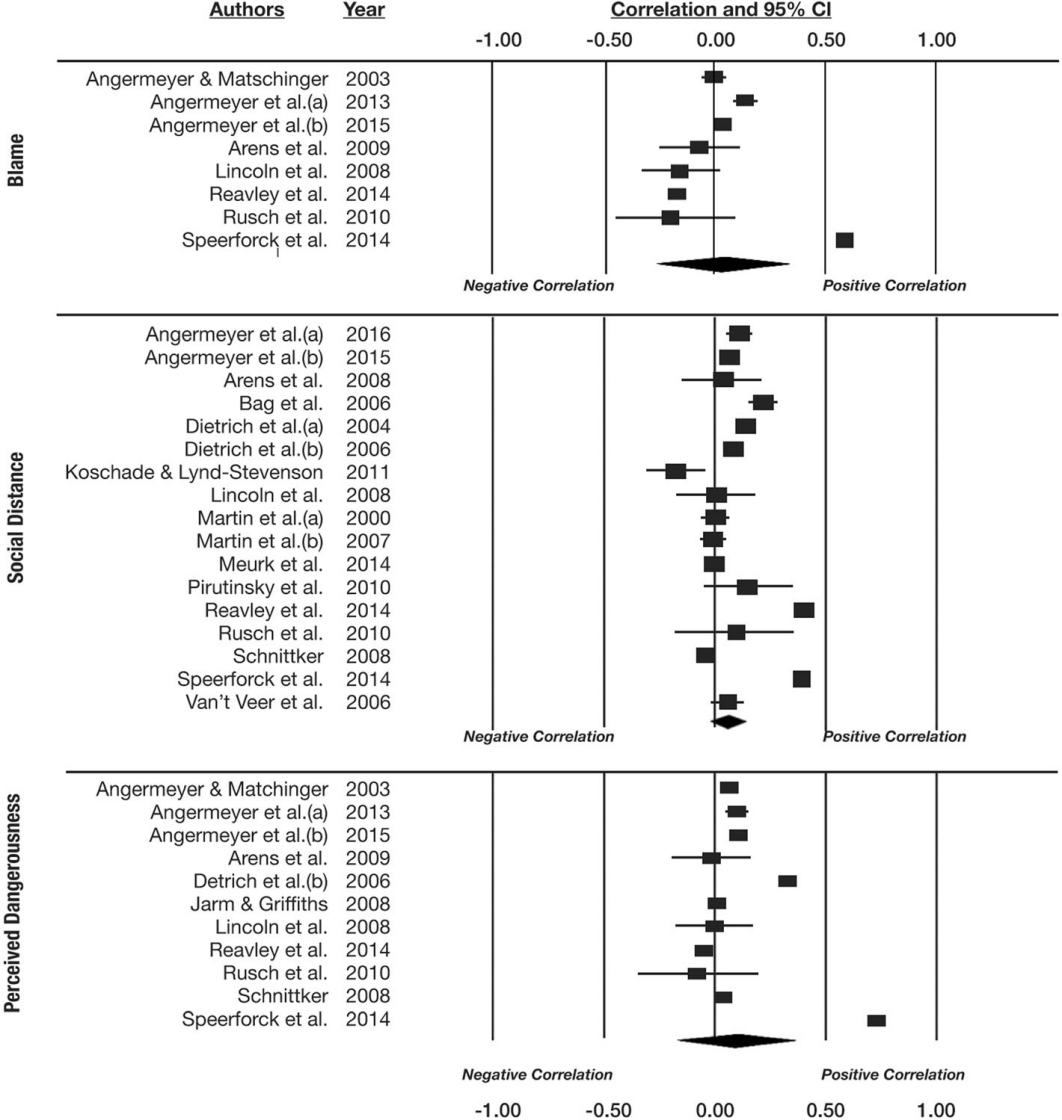
Plots show meta-analyses of the correlational studies

The second set of meta-analyses synthesized the 19 correlational studies. There were 9 studies that yielded effect seizes for the blame component of stigma, 17 for social distance, and 11 for perceived dangerousness. No studies investigated associations with the prognostic pessimism component of stigma. The findings are presented in Fig. 3 and Table 3. Although these meta-analyses contained more studies than the experimental meta-analyses, in contrast to those analyses, none of them yielded aggregate effects that reached conventional levels of significance. Endorsement of neurobiological explanations was not associated with greater or lesser blame (*r* = .039, *p* = .804), but it was marginally associated with a greater desire for social distance (*r* = .101, *p* = .058), and non-significantly associated with greater perceived dangerousness (*r* = .138, *p* = .257). None of these conclusions was reliably qualified by psychiatric condition, although relatively few studies have examined any specific condition, especially those other than depression and schizophrenia. Careful inspection of the study by Speerforck, Schomerus, Pruess, and Angermeyer (2014), which generated two outlying effects, failed to yield an explanation of why the effects might be spurious. As both meta-analytic effects were non-significant and weakly trending in the direction of the outliers, removal of those outliers, which is contrary to the spirit of meta-analysis, would not have altered the overall findings.

**Table 3:** Summary of meta-analytic findings from correlational studies

Stigma component	Condition	Number of studies	*r* estimate	95% CI - lower	95% CI - upper	*p*
Blame						
	Overall	8	.039	−.260	.331	.804
	Depression	3	.276	−.206	.650	.260
	Schizophrenia	7	−.001	−.297	.294	.994
	Other	2	.016	−.223	.253	.895
Social distance						
	Overall	17	.101	−.003	.202	.058
	Depression	2	.084	.059	.110	< 0.001
	Schizophrenia	5	.169	.145	.192	< 0.001
	Other	12	.336	.329	.344	< 0.001
Dangerousness						
	Overall	11	.138	−.101	.361	.257
	Depression	6	.228	−.100	.511	.172
	Schizophrenia	8	.169	−.124	.436	.258
	Other	4	.057	.089	.089	< 0.001

## Discussion

The findings of the present study were broadly consistent internally and with previous meta-analyses. Internally, the meta-analyses of correlational and experimental studies yielded similar patterns of findings. Neurobiological explanations were positively associated with desire for social distance in both meta-analyses (only marginally for the correlational analysis) and they were unrelated to blame in both meta-analyses. Neurobiological explanations were significantly associated with greater perceived dangerousness in the experimental meta-analysis and although there was no significant relationship in the correlational meta-analysis the trend was in the same direction. As there were no correlational studies of prognostic pessimism, consistency with the significant positive relationship between neurobiological explanation and this stigma dimension could not be assessed. Despite the overall consistency, it must also be noted that the experimental meta-analyses yielded much stronger evidence for relationships between neurobiological explanation and stigma than the correlational meta-analyses, despite including fewer primary studies. This difference may reflect the many confounding factors and moderator variables present in correlational studies of stigma (e.g., Kvaale & Haslam, 2016) that may hamper the detection of relationships. In view of the superiority of experimental designs in detecting causal relationships, the stronger evidence for the adverse effects of neurobiological explanation on stigma in the experimental meta-analyses is a reason for confidence in those effects.

The findings also accord well with the prior investigations of Kvaale, Gottdiener, and Haslam (2013), and Kvaale, Haslam, and Gottdiener (2013), whose focus was on the broader set of biogenetic explanations (i.e., including genetic explanations in addition to neurobiological explanations). The only discrepancies between past and present meta-analyses point to somewhat more adverse implications of neurobiological explanations in the present study. Biogenetic explanations were associated with reduced blame in Kvaale, Gottdiener, and Haslam (2013) and Kvaale, Haslam, and Gottdiener (2013), but neurobiological explanations had a null relationship with blame in the present analyses. Kvaale, Haslam, and Gottdiener (2013) found a null relationship between biogenetic explanations and desire for social distance in experimental studies, but the present study found a positive relationship in such studies, which provide the best evidence that neurobiological explanations have a causative effect in psychiatric stigma.

All in all, the present study suggests that neurobiological explanations are at least as strongly associated with psychiatric stigma as with biogenetic explanations more generally. Although it is theoretically plausible that laypeople’s neurobiological explanations might be less essentialistic than their genetic explanations – less deep-seated, less discrete, and less fixed – these neurobiological explanations appear to be linked to multiple components of stigma in ways that are every bit as problematic as genetic explanations, if not more so. Although much of the concern over the effect of biogenetic explanation in psychiatric stigma has targeted genetic explanation and genetic essentialism (Dar-Nimrod & Heine, 2011a; Phelan, 2005), neurobiological explanation and neuroessentialism may be just as concerning.

Three explanations might be offered for particular aspects of the findings. First, it could be argued that the failure to detect significant aggregate effects in some analyses at least partially reflects low statistical power. Because neurobiological explanations are a subset of biogenetic explanations, analyses restricted to the former may not have been powerful enough to detect the absence of negative relationships between neurobiological explanations and blame, for example. It is true that fewer studies were included in the present experimental meta-analysis than in Kvaale, Haslam, and Gottdiener (2013) – a mean of 4.0 studies per analysis, compared to 13.75 – and this may have prevented the trend for blame to be negatively related to neurobiological explanations to reach significance, as it did in the earlier meta-analysis. However, the present analysis of correlational findings pertaining to blame synthesized *more* effect sizes than the corresponding analysis in Kvaale, Gottdiener, and Haslam (2013), and not only failed to replicate that significant negative relationship in the study but obtained an overall effect size that was non-significantly *positive*. The finding that neurobiological explanations were not negatively associated with blame therefore cannot be fully attributed to low statistical power.

A second explanation addresses the pattern of adverse links between neurobiological explanations and stigma obtained in the present study. The consistency of this pattern is perhaps surprising, given that neurobiological explanations might seem to be less essentialist than some other biogenetic explanations, such as those involving genetic causes. Arguably neurobiological explanations are not, in fact, less likely than genetic or other biological explanations to invoke essentialist thinking among the lay public. Neuroscientists may have nonessentialist understanding of the brain mechanisms involved in mental health problems, recognizing that these mechanisms are not discrete but continuous with normality; that they are not localized as pathological essences but distributed in complex networks; and that they are intrinsically plastic rather than fixed and deterministic. However, laypeople may understand the brain differently: as a mysterious seat of the soul whose abnormalities determine the person’s fate and identity. They may think of the neural bases of mental disorders as discrete and localized lesions, perhaps encouraged by the tendency for media reports of neuroscientific findings to invoke specific brain regions. When they understand depression or schizophrenia as “brain diseases” they may liken these conditions to degenerative diseases such as dementia, which do have a pessimistic prognosis and seem to determine and disrupt personal identity, or to traumatic conditions that are equally deep and enduring in their effects. Similarly, when laypeople endorse “chemical imbalances” as explanations of mental ill-health they may apprehend them as deep-seated and enduring defects that can be palliated by medication but never cured. Thus, even if the neuroscientific explanations for mental disorder that are generated by researchers are not intrinsically essentialist, when they are refracted through the lens of psychological essentialism by laypeople they may become so. As a result, neurobiological explanations may come to have adverse implications for psychiatric stigma, contrary to the destigmatizing intentions of neuroscience researchers.

A final explanation for the consistent relationships between neurobiological explanations and stigma is that these explanations are not in fact highly essentialistic and are associated with stigma for different reasons. By this account, laypeople may hold negative beliefs about people whose problems are given neurobiological explanations for reasons that have little to do with the hidden, deep-seated, or fixed nature of the supposed causes. Neurobiological explanations may elicit desire for social distance and fear, for example, because people believe these explanations imply that the affected person is not in control of her own actions. If the brain is understood to be the source of free will and agency, then any explanation that calls into question its integrity may be interpreted as a sign that the person may behave in unpredictable, uncontrollable, or dangerous ways. The links between neurobiological explanations and stigma found in the present study may therefore point to specific, control-related elements of these explanations that may not be shared with other kinds of biogenetic explanation. Further research is need to ascertain the degree to which links between neurobiological explanation and stigma are grounded in essentialist thinking.

The present meta-analyses have some limitations. First, the number of studies included is relatively modest, limiting the power of some statistical tests of aggregate effect sizes. This limitation is only partial, with three of the seven effects examined reaching conventional levels of statistical significance, and it is likely to be overcome in future as additional studies accumulate. Second, and relatedly, study numbers were too small to allow us to examine whether effects vary systematically across different populations (e.g., healthy participants, people experiencing mental disorders, mental health professionals). Third, all of the experimental studies included in our meta-analyses compared neurobiological explanations to psychosocial explanations, making it difficult to infer the direct rather than comparative influence of neurobiological explanation on stigma. Future research should attempt to disentangle these two forms of explanation. Experimental studies should also aim to directly compare the effects on stigma of different kinds of biogenetic explanation.

## Conclusions

Our findings indicate that explanations of mental health problems that invoke the brain have some problematic implications for the public’s attitudes towards people who experience them. People who tend to explain psychiatric conditions as brain diseases or as products of chemical imbalances may be especially likely to avoid sufferers and are just as likely as others to blame them for their problems. People who are given a neurobiological explanation of a psychiatric condition tend to see sufferers as more dangerous and less likely to recover, and are more likely to distance themselves from them, than people who are not. Neuroscientific explanations might appear to hold promise as ways to destigmatize mental health problems, representing them as biomedical illnesses rather than as personal weaknesses or taints, but the public reception of them – and perhaps their reception by professionals as well (Lebowitz & Ahn, 2014) – sometimes seems to have the opposite effect.

This troubling conclusion has implications for how the findings of psychiatric neuroscience should be communicated. Such findings are seductive (Weisberg et al., 2008) and they are also prone to be apprehended in biased ways. Mindful that neurobiological explanations may be misunderstood in an essentialist fashion – as picking out a discrete, unalterable, and identity-determining defect – neuroscientists should take pains to counteract rather than play into these neuroessentialist distortions. Where possible, discussions of the neurobiological dimensions of mental health problems should avoid the sort of reductive simplification that presents “brain disease” or “chemical imbalance” as a static essence. The rise of psychiatric neuroscience is unlikely to erode stigma unless it is communicated to the public in a way that emphasizes complexity over reduction and plasticity over fixity.

## Appendix

### Search terms

**MeSh terms**

‘Social stigma’ OR ‘social distance’ OR ‘fear’ OR ‘scapegoating’ OR ‘anger’ OR ‘prognosis’

AND ‘Genetics, Behavioral’

AND ‘Mental Disorders’

Combined Mesh and Keyword search terms in Pubmed (#44 AND #45 AND #46) = 1775 results

*((((("mental disorders"[MeSH Terms] OR ("mental"[All Fields] AND "disorders"[All Fields]) OR "mental disorders"[All Fields] OR ("mental"[All Fields] AND "disorder"[All Fields]) OR "mental disorder"[All Fields]) OR ("mental disorders"[MeSH Terms] OR ("mental"[All Fields] AND "disorders"[All Fields]) OR "mental disorders"[All Fields] OR ("mental"[All Fields] AND "illness"[All Fields]) OR "mental illness"[All Fields])) OR "mental disorders"[MeSH Terms]) AND ("2011/01/01"[PDAT] : "2018/12/31"[PDAT]) AND "humans"[MeSH Terms] AND English[lang]) AND ((("biology"[MeSH Terms] OR "biology"[All Fields] OR "biological"[All Fields]) OR biogenetic[All Fields] OR ("genetic therapy"[MeSH Terms] OR ("genetic"[All Fields] AND "therapy"[All Fields]) OR "genetic therapy"[All Fields] OR "genetic"[All Fields])) AND ("2011/01/01"[PDAT] : "2018/12/31"[PDAT]) AND "humans"[MeSH Terms] AND English[lang])) AND (((((((("prognosis"[MeSH Terms] OR "anger"[MeSH Terms]) OR "fear"[MeSH Terms]) OR "scapegoating"[MeSH Terms]) OR "social stigma"[MeSH Terms]) OR "social distance"[MeSH Terms]) AND ("2011/01/01"[PDAT] : "2018/12/31"[PDAT]) AND "humans"[MeSH Terms] AND English[lang]) OR (("social stigma"[MeSH Terms] OR ("social"[All Fields] AND "stigma"[All Fields]) OR "social stigma"[All Fields] OR "stigma"[All Fields]) OR ("social distance"[MeSH Terms] OR ("social"[All Fields] AND "distance"[All Fields]) OR "social distance"[All Fields]) OR (danger[All Fields] OR danger'[All Fields] OR danger's[All Fields] OR danger1a[All Fields] OR danger associated[All Fields] OR dangered[All Fields] OR dangereous[All Fields] OR dangereuse[All Fields] OR dangereusement[All Fields] OR dangereuses[All Fields] OR dangereuses'[All Fields] OR dangereux[All Fields] OR dangerfield[All Fields] OR dangergregoire[All Fields] OR dangerless[All Fields] OR dangerma[All Fields] OR dangerman[All Fields] OR dangerosit'e[All Fields] OR dangerosite[All Fields] OR dangerosity[All Fields] OR dangerous[All Fields] OR dangerous'[All Fields] OR dangerous"[All Fields] OR dangerousfrizbee[All Fields] OR dangerousity[All Fields] OR dangerousless[All Fields] OR dangerously[All Fields] OR dangerously'[All Fields] OR dangerousness[All Fields] OR dangerousness'[All Fields] OR dangeroussness[All Fields] OR dangerousy[All Fields] OR dangers[All Fields] OR dangers'[All Fields] OR dangersofcesareanbirth[All Fields] OR dangerzone[All Fields]) OR ("fear"[MeSH Terms] OR "fear"[All Fields]) OR blame[All Fields] OR ("anger"[MeSH Terms] OR "anger"[All Fields]) OR hopelessness[All Fields] OR (("poverty"[MeSH Terms] OR "poverty"[All Fields] OR "poor"[All Fields]) AND ("prognosis"[MeSH Terms] OR "prognosis"[All Fields])))) AND ("2011/01/01"[PDAT] : "2018/12/31"[PDAT]) AND "humans"[MeSH Terms] AND English[lang]) AND (("2011/01/01"[PDAT] : "2018/12/31"[PDAT]) AND "humans"[MeSH Terms] AND English[lang])*.

**SCOPUS search terms**

( TITLE-ABS-KEY ( biological OR biogenetic OR genetic ) ) AND ( TITLE-ABS-KEY ( "mental disorder" OR "mental illness" ) ) AND ( TITLE-ABS-KEY ( stigma OR "social distance" OR danger* OR fear OR blame OR anger OR hopelessness OR "poor prognosis" ) ) AND ( LIMIT-TO ( PUBYEAR , 2017 ) OR LIMIT-TO ( PUBYEAR , 2016 ) OR LIMIT-TO ( PUBYEAR , 2015 ) OR LIMIT-TO ( PUBYEAR , 2014 ) OR LIMIT-TO ( PUBYEAR , 2013 ) OR LIMIT-TO ( PUBYEAR , 2012 ) OR LIMIT-TO ( PUBYEAR , 2011 ) ) AND ( LIMIT-TO ( DOCTYPE , "ar" ) OR LIMIT-TO ( DOCTYPE , "re" ) OR LIMIT-TO ( DOCTYPE , "ch" ) OR LIMIT-TO ( DOCTYPE , "ed" ) OR LIMIT-TO ( DOCTYPE , "sh" ) OR LIMIT-TO ( DOCTYPE , "ip" ) OR LIMIT-TO ( DOCTYPE , "no" ) OR LIMIT-TO ( DOCTYPE , "le" ) OR LIMIT-TO ( DOCTYPE , "er" ) ) AND ( LIMIT-TO ( LANGUAGE , "English" ) ).

## Footnotes

1 We use the term “neurobiological explanation” to refer to explanations invoking causes that explicitly or implicitly implicate the nervous system (e.g., brain disease or dysfunction, neurochemistry). This usage includes explanations endorsed by laypeople that lack specificity or scientific basis (e.g., “chemical imbalance”). In contrast, we refer to “neuroscientific explanations” to refer to explanations involving neurobiological causes that are propounded by neuroscientists. We use the expression “biogenetic explanation” as an umbrella term that encompasses many forms of biological explanation, including neurobiological explanation as one form but also including explanations invoking genetic influences and unspecified biological explanations that are neither neurobiological nor genetic.
